# Analysis of Plasma Protein Concentrations and Enzyme Activities in Cattle within the Ex-Evacuation Zone of the Fukushima Daiichi Nuclear Plant Accident

**DOI:** 10.1371/journal.pone.0155069

**Published:** 2016-05-09

**Authors:** Yusuke Urushihara, Koh Kawasumi, Satoru Endo, Kenichi Tanaka, Yasuko Hirakawa, Gohei Hayashi, Tsutomu Sekine, Yasushi Kino, Yoshikazu Kuwahara, Masatoshi Suzuki, Motoi Fukumoto, Hideaki Yamashiro, Yasuyuki Abe, Tomokazu Fukuda, Hisashi Shinoda, Emiko Isogai, Toshiro Arai, Manabu Fukumoto

**Affiliations:** 1 Institute of Development, Aging and Cancer, Tohoku University, Sendai, Miyagi, Japan; 2 Fukushima Project Headquarters, National Institute of Radiological Sciences, Chiba, Chiba, Japan; 3 School of Veterinary Medicine, Nippon Veterinary and Life Science University, Musashino, Tokyo, Japan; 4 Graduate School of Engineering, Hiroshima University, Higashi-Hiroshima, Hiroshima, Japan; 5 Institute for Excellence in Higher Education, Tohoku University, Sendai, Miyagi, Japan; 6 Graduate School of Science, Tohoku University, Sendai, Miyagi, Japan; 7 Faculty of Medicine, Tohoku Medical and Pharmaceutical University, Sendai, Miyagi, Japan; 8 Faculty of Agriculture, Niigata University, Niigata, Niigata, Japan; 9 National Research Center for Protozoan Diseases, Obihiro University of Agriculture and Veterinary Medicine, Obihiro, Hokkaido, Japan; 10 Graduate School of Agricultural Sciences, Tohoku University, Sendai, Miyagi, Japan; 11 Graduate School of Dentistry, Tohoku University, Sendai, Miyagi, Japan; University of South Carolina, UNITED STATES

## Abstract

The effect of the Fukushima Daiichi Nuclear Power Plant (FNPP) accident on humans and the environment is a global concern. We performed biochemical analyses of plasma from 49 Japanese Black cattle that were euthanized in the ex-evacuation zone set within a 20-km radius of FNPP. Among radionuclides attributable to the FNPP accident, germanium gamma-ray spectrometry detected photopeaks only from ^134^Cs and ^137^Cs (radiocesium) commonly in the organs and in soil examined. Radioactivity concentration of radiocesium was the highest in skeletal muscles. Assuming that the animal body was composed of only skeletal muscles, the median of internal dose rate from radiocesium was 12.5 μGy/day (ranging from 1.6 to 33.9 μGy/day). The median of external dose rate calculating from the place the cattle were caught was 18.8 μGy/day (6.0–133.4 μGy/day). The median of internal and external (total) dose rate of the individual cattle was 26.9 μGy/day (9.1–155.1 μGy/day). Plasma levels of malondialdehyde and superoxide dismutase activity were positively and glutathione peroxidase activity was negatively correlated with internal dose rate. Plasma alanine transaminase activity and percent activity of lactate dehydrogenase (LDH)-2, LDH-3 and LDH-4 were positively and LDH-1 was negatively correlated with both internal and total dose rate. These suggest that chronic exposure to low-dose rate of ionizing radiation induces slight stress resulting in modified plasma protein and enzyme levels.

## Introduction

A large amount of radionuclides were released from Fukushima Daiichi Nuclear Power Plant (FNPP) due to the Great East Japan Earthquake and the subsequent tsunami [1]. Because the effect of long-term exposure to low-dose rate radiation (LDRR) remains to be elucidated, worldwide attention has been focused on the impact of the FNPP accident on humans and the environment [2–4]. The residents within the area of a 20-km radius around FNPP were recommended to evacuate on March 12 and the Japanese government set the area as the evacuation zone based on the 20-km radius around FNPP on April 22, 2011. The Japanese government ordered the governor of Fukushima prefecture to euthanize abandoned livestock within the zone on May 12, 2011. Since April 2012, rearrangements of the restricted areas, including the evacuation zone, have been performed. We, therefore, term the area within a 20-km radius of FNPP the ex-evacuation zone. It has been reported that livestock residing within the ex-evacuation zone were exposed to internally deposited radionuclides and external radiation [5–7]. Biological impacts occurred after the FNPP accident have been reported [8–10], however the association of those with the FNPP accident remains uncertain because of the difficulty of the estimation of radiation dose.

It is necessary to identify biomarkers of exposure to LDRR and the biological effect in animals for establishing radiation protection in humans. The levels of plasma proteins and enzyme activities are routinely measured to monitor health conditions in cattle as well as in humans.

Reactive oxygen species (ROS) are generated by the interaction between water molecules and radiation. ROS are scavenged by antioxidant enzymes such as superoxide dismutase (SOD) and glutathione peroxidase (GPx). SOD catalyzes the dismutation of superoxide radical anion (O_2_^-^) to form H_2_O_2_ and molecular oxygen (O_2_), and GPx and catalase catalyze the reduction of H_2_O_2_ to H_2_O. Low-dose radiation induces defensive responses such as the detoxification of ROS [11]. Continuous LDRR to endothelial cells *in vitro* induces senescence triggered by the stress response [12, 13]. Oxidative stress parameters in radiology staff who were occupationally exposed to ionizing radiation in a hospital have been evaluated. Compared with the control subjects, the activities of erythrocyte CuZn-SOD and Se-GPx are significantly higher, and catalase activity and the levels of a lipid peroxidation product, malondialdehyde (MDA) are lower in the exposed group [14]. The erythrocyte activities of SOD, catalase and GPx are higher in workers of X-ray departments compared with control workers [15]. The levels of an antioxidant, glutathione in mononuclear blood cells of radiology technicians are lower than those of the control group [16]. These findings prompted us to postulate that chronic exposure to low-dose radiation might increase oxidative stress in livestock within the ex-evacuation zone of the FNPP accident.

To assess the contamination of the ecosystem and the biological effect following the FNPP accident, we recently established an archive system composed of organs of livestock and wild animals within and around the ex-evacuation zone [17]. We have been performing sampling of organs from cattle since 5 months after the FNPP accident. Almost all short lived radionuclides released from FNPP had already decayed out (S1 Fig). Therefore, ^134^Cs and ^137^Cs (radiocesium) have been a major concern because they have been the only radionuclides detected in all the organs and in soil examined in this study at the same radioactivity concentration as of the days of their release [5], and have deleterious effects on the environment due to their long half-lives [18]. In the present study, we calculated internal dose rate from radiocesium concentration in skeletal muscles and external dose rate from that in soil. We then sought to identify plasma biomarkers of exposure to chronic LDRR from radiocesium.

## Materials and Methods

### Ethics

This study is one of the national projects associated with the Great East Japan Earthquake and has been entirely endorsed and supported by the Japanese government through the Ministry of Education, Culture, Sports, Science and Technology, Japan. The Japanese government ordered the Fukushima prefecture to euthanize livestock in the ex-evacuation zone on May 12, 2011 to prevent contaminated meat from becoming the potential source of radiation by entering the food chain. We obtained organs from the cattle euthanized by the combined unit of veterinarians belonging to the Livestock Hygiene Service Centre (LHSC) of Fukushima prefecture and to the Ministry of Agriculture, Forestry and Fisheries, Japan. Euthanasia was performed according to the Regulation for Animal Experiments and Related Activities at Tohoku University (Regulation No 122). Informed consent from the owners was obtained by the veterinarians of LHSC. This study was approved by the Ethics Committee of Animal Experiments, Tohoku University (2014-IDAC-037).

### Animals

Cattle were abandoned and unleashed since March 12, 2011 and were free to move and access the contaminated grass in the ex-evacuation zone including formerly residential areas until euthanasia. We performed sampling from 49 euthanized cattle in the ex-evacuation zone from August 29, 2011 to August 10, 2012. All the cattle were adults of Japanese Black consisting of 40 non-pregnant and 5 pregnant females, 2 non-castrated bulls and 2 castrated oxen. As controls without the effect of the FNPP accident, we collected peripheral blood from 6 Japanese black cattle in the Miyagi prefectural farm at Iwadeyama (130 km north of FNPP) in March 2013 and 4 Japanese black cattle in a farm of Yamaguchi prefecture (950 km southeast of FNPP) in April 2013.

### Blood and skeletal muscle sampling

Heparinized peripheral blood was collected from the jugular vein and was immediately centrifuged to separate plasma and blood cells. Plasma was preserved at -80°C until use. Skeletal muscle samples were obtained from two representative positions: the longissimus and bicep femoris muscles.

### Biochemical and enzyme activity analyses of plasma

Plasma biochemistry analyses of glucose (GLU), total cholesterol (TC), triglycerides (TG), aspartate aminotransferase (AST), alanine aminotransferase (ALT), alkaline phosphatase (ALP), lactate dehydrogenase (LDH), total protein (TP), blood urea nitrogen (BUN), and creatinine (CRE) were performed using an autoanalyzer (JCA-BM2250, JEOL Ltd., Tokyo, Japan) according to the manufacturer’s protocol. The plasma non-esterified fatty acid (NEFA) concentration was measured using a Wako NEFA-C test commercial kit (Wako Pure Chemical Industries, Inc., Tokyo, Japan). Commercial kits were used for measurement of plasma MDA concentration (NWK-MDA01), SOD activity (NWK-SOD01) and GPx activity (NWK-GPX01, Northwest Life Science Specialties, LLC, Vancouver, Canada). Plasma LDH isozyme pattern was confirmed by a QuickGel LD Isoenzyme kit (Helena Laboratories, Texas, USA) with an Edbank III analysis software (Helena Laboratories, Texas, USA).

### Measurement of radioactivity

Radioactivity was determined by gamma-ray spectrometry using high-purity Germanium (HPGe) detectors (GEM40P4-83, Ortec Co., Oak Ridge, TN, USA) as described previously [5]. In brief, duration of the measurement varied from 3,600 to 86,400 seconds, depending on the radioactivity of the sample. Standard volume sources having a different size were prepared by diluting stock solutions of Cs-137 and Eu-152 and gelling with superabsorbent polymer. Activities of the stock solutions were calibrated with standard point sources of Cs-137 (10 kBq, CS402) and Eu-152 (10 kBq, EU402, Japan Isotope Association, Tokyo, Japan). Samples were homogenized and scaled in polyethylene vials. A nuclide was identified when its characteristic photopeaks of greater than 3σabove the baseline were observed in the spectrum.

### Estimation of internal radiation dose rate

Radioactivity of organ samples was determined by gamma-ray spectrometry. In the spectra of all the organs examined, photopeaks commonly attributable to the FNPP accident were only from radiocesium (S1 Fig) [5]. To calculate dose rate for internal exposure to gamma and beta rays from radiocesium, we used the dose conversion coefficients of the reference deer adopted by International Commission on Radiological Protection (ICRP): 1.5×10^−2^ [(μGy/day)/(Bq/kg)] for ^134^Cs and 8.2×10^−3^ [(μGy/day)/(Bq/kg)] for ^137^Cs [19]. We assumed that the whole body of a cattle was composed of only the skeletal muscle because radiocesium concentration is the highest in the skeletal muscle among the organs measured [5] and the whole body is covered with the skeletal muscle. In some cattle, only peripheral blood but not organ specimens was obtained. In those cases, we converted the radiocesium concentration in the blood to the muscle using the conversion factor 27.8 (S2 Fig and S1 Table), which is consistent with our previous report [20].

### Estimation of external radiation dose rate

External dose rate was calculated from deposited radiocesium in the soil where the cattle were captured (S1 Table). Using the 2-km mesh soil contamination measurements [21], we drew a radiocesium pollution map in the ex-evacuation zone with a geographic information system computer program, System for Automated Geoscientific Analyses (SAGA GIS) [22]. We interpolated radiocaesum concentration at the sampling sites as the same method as recently reported [23]. We calculated external dose rate from radiocesium concentration in soil using the dose conversion coefficients of the reference deer by ICRP, 6.1×10^−5^ [(μGy/day)/(Bq/m^2^)] for ^134^Cs and 2.2×10^−5^ [(μGy/day)/(Bq/ m^2^)] for ^137^Cs [18].

### Statistical analysis

Pearson’s correlation coefficient was used to examine correlations between dose rate and cumulative dose from radiocesium, and plasma components. Werch’s t test was applied to determine significant differences between 2 groups.

## Results

### Plasma protein concentrations and enzyme activities

**Fig 1:**
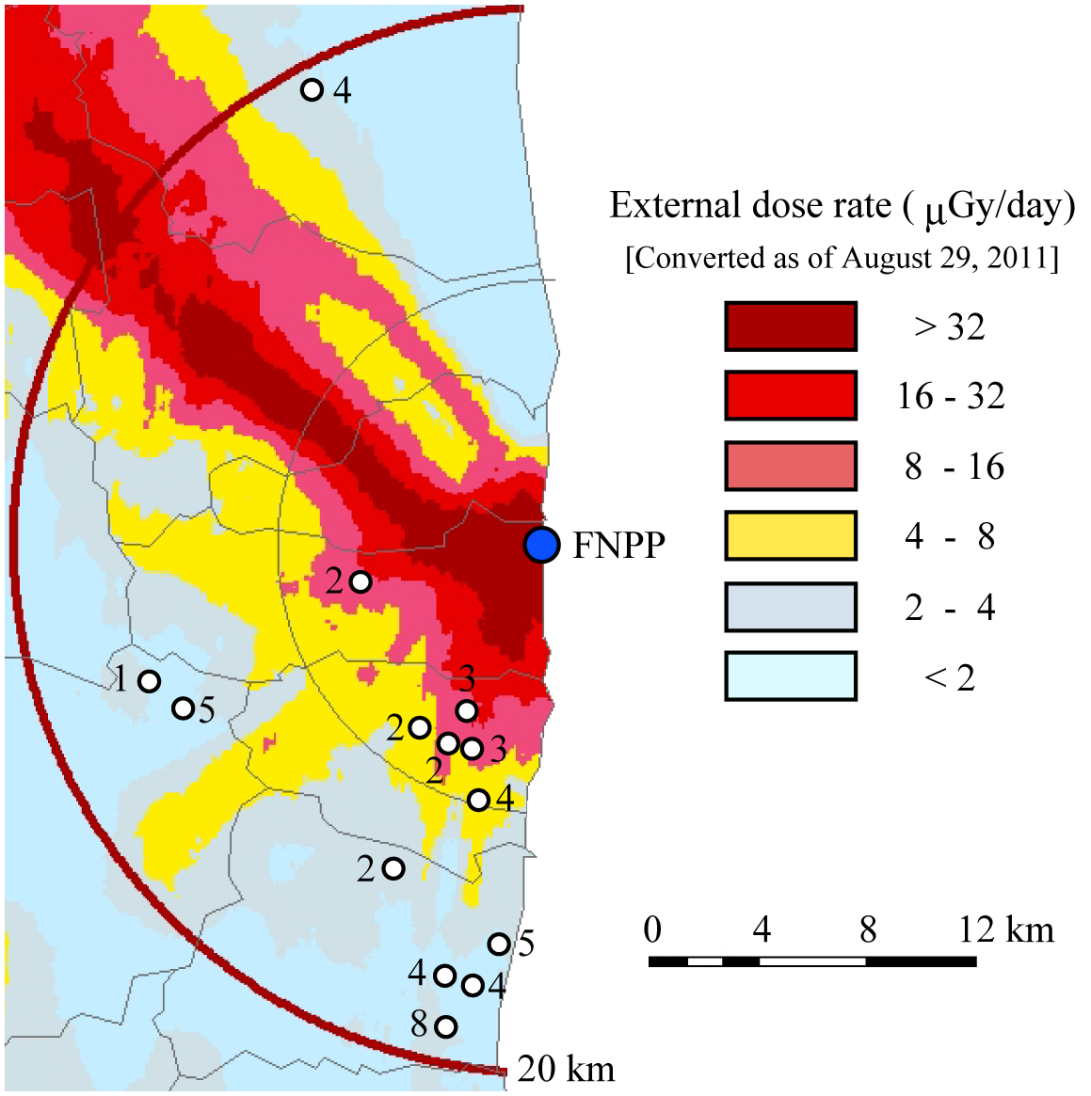
Map showing the sampling point. A blue circle indicates the location of Fukushima Daiichi Nuclear Power Plant. White circles indicate the sampling point of cattle. The number indicates the number of cattle captured on each site. Modified from the 2-km mesh soil contamination measurement [21] using a System for Automated Geoscientific Analyses (SAGA) geographic information system (GIS) computer program [22].

Plasma protein concentrations and enzyme activities measured in this study are shown in Table 1. We obtained peripheral blood from 49 Japanese Back cattle at 14 sampling sites in the ex-evacuation zone (Fig 1). We measured the levels of TP, TG, BUN, CRE, TC, GLU, and NEFA, as well as the activities of enzymes AST, ALT, ALP, LDH, LDH isozymes, SOD and GPx in the plasma. We also measured the concentration of the oxidative stress marker, MDA (Table 1). Plasma components in cattle can be affected by the age and the breeding condition [24]. Although reference values of blood biochemical data are assumed to be dependent on the strain and the feeding condition, available data of Japanese Black have not been opened. We therefore, determined biochemical values in Japanese Black cattle at the Miyagi prefectural farm (130 km north of FNPP) and a farm in Yamaguchi prefecture (950 km southeast of FNPP) as the control not affected by the FNPP accident. TP, ALP, total LDH, LDH-1, LDH-3, LDH-4, CRE, TC, MDA and GPx were significantly different between Miyagi group and Yamaguchi group. TP, ALP, LDH-1, LDH-2, LDH-3, CRE, NEFA, MDA, SOD and GPx in the cattle of the ex-evacuation zone were significantly different from both those of Miyagi group and Yamaguchi group, respectively.

**Table 1:** Plasma protein concentrations and enzyme activities.

	Ex-evacuation (49)	Miyagi (6)	Yamaguchi (4)
Gender	43 females and 4 males^#^	6 males^##^	1 female and 3 males^##^
Age (year)*	6.36 ± 3.26^†^	0.69 ± 0.02	1.71 ± 0.21
TP (g/dL)*	6.96 ± 0.85^†^	6.23 ± 0.43	7.40 ± 0.28
TG (mg/dL)	13.6 ± 9.7	11.3 ± 3.1	19.3 ± 5.4
AST (IU/L)	72.9 ± 24.7	55.8 ± 5.9	86.8 ± 21.9
ALT (IU/L)	16.5 ± 5.8	23.3 ± 2.1	22.8 ± 5.1
ALP (IU/L)*	157.0 ± 122.9^†^	571.5 ± 192.3	284.0 ± 76.6
LDH (IU/L)*	1006.1 ± 256.1	953.5 ± 66.4	1492.3 ± 113.5
LDH-1 (%)*	50.8 ± 7.4^†^	41.6 ± 0.8	47.2 ± 1.6
LDH-2 (%)	25.7 ± 2.4^†^	29.3 ± 1.1	28.6 ± 1.2
LDH-3 (%)*	14.5 ± 3.4^†^	18.6 ± 0.6	16.8 ± 0.7
LDH-4 (%)*	5.3 ± 1.8	6.6 ± 0.6	4.8 ± 0.9
LDH-5 (%)	3.9 ± 2.0	4.0 ± 0.9	2.7 ± 1.1
BUN (mg/dL)	8.0 ± 4.9	12.5 ± 2.1	12.3 ± 4.9
CRE (mg/dL)*	1.34 ± 0.26^†^	0.78 ± 0.10	1.2 ± 0.00
TC (mg/dL)*	91.5 ± 35.9	78.0 ± 20.5	125.5 ± 26.0
GLU (mg/dL)	88.1 ± 67.0	89.8 ± 4.7	103.0 ± 46.3
NEFA (μEq/L)	308 ± 192^†^	140 ± 79	87 ± 5
MDA (μmol/L)*	2.19 ± 0.94^†^	0.83 ± 0.09	1.08 ± 0.13
SOD (U/mL)	12.7 ± 14.1	45.0 ± 34.3	25.8 ± 6.7
GPx (mU/mL)*	10.4 ± 3.9^†^	6.4 ± 3.5	51.6 ± 9.5

The numbers in parentheses indicate the number of animals examined.

^#^Two were castrated.

^##^All the males were castrated.

*Significantly different between Miyagi and Yamaguchi groups (p < 0.05).

^†^Significantly higher or lower than both Miyagi and Yamaguchi groups (p < 0.05).

### Dose rate and plasma components

**Fig 2:**
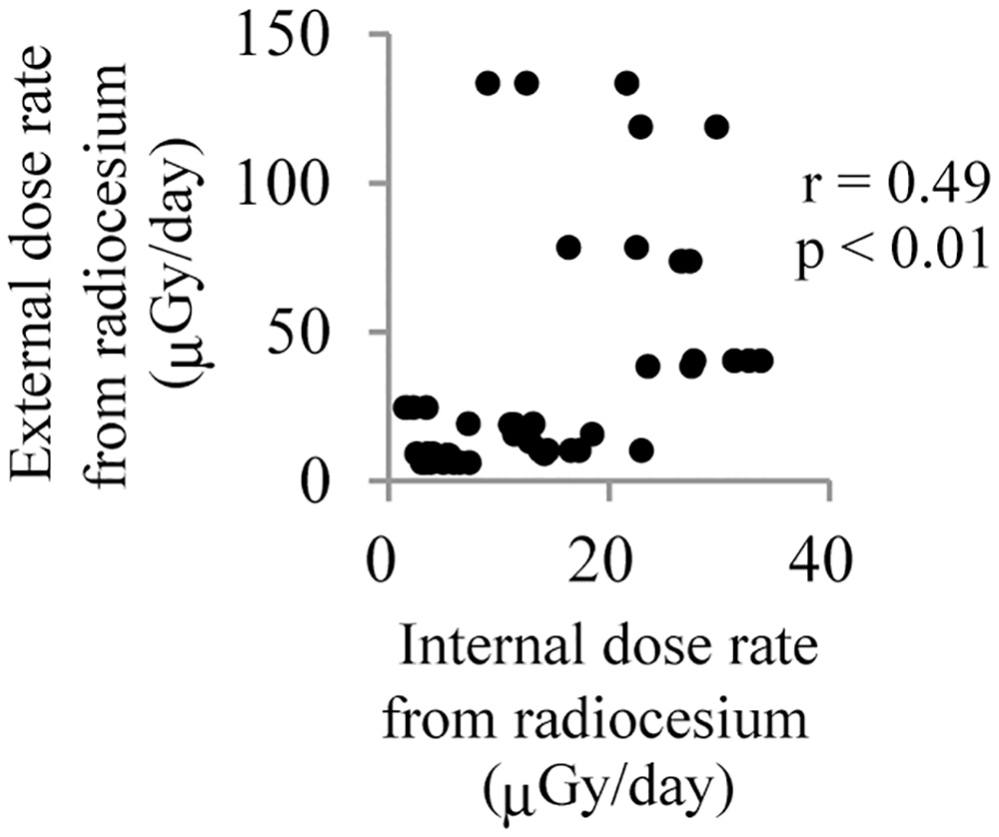
Correlation analysis between internal dose rate and external dose rate. The dot plot shows the correlation of internal dose rate with external dose rate in cattle in the ex-evacuation zone. r and p is Pearson’s correlation coefficient and p value, respectively.

We calculated dose rate of internal exposure from the radiocesium concentration in the skeletal muscle and that of external exposure from the radiocesium concentration in soil. The dispersion of dose rate of individual cattle was wider from external exposure than from internal exposure (Fig 2). The median of internal dose rate was 12.5 μGy/day ranging from 1.6 to 33.9 μGy/day, and those of external dose rate were 18.8 μGy/day, ranging 6.0 to133.4 μGy/day. Correlation between internal dose rate and external dose rate was moderate (r = 0.47, p < 0.001) (Fig 2). The median of internal and external (total) dose rate of the individual cattle was 26.9 μGy/day, ranging from 9.1 to 155.1 μGy/day.

**Fig 3:**
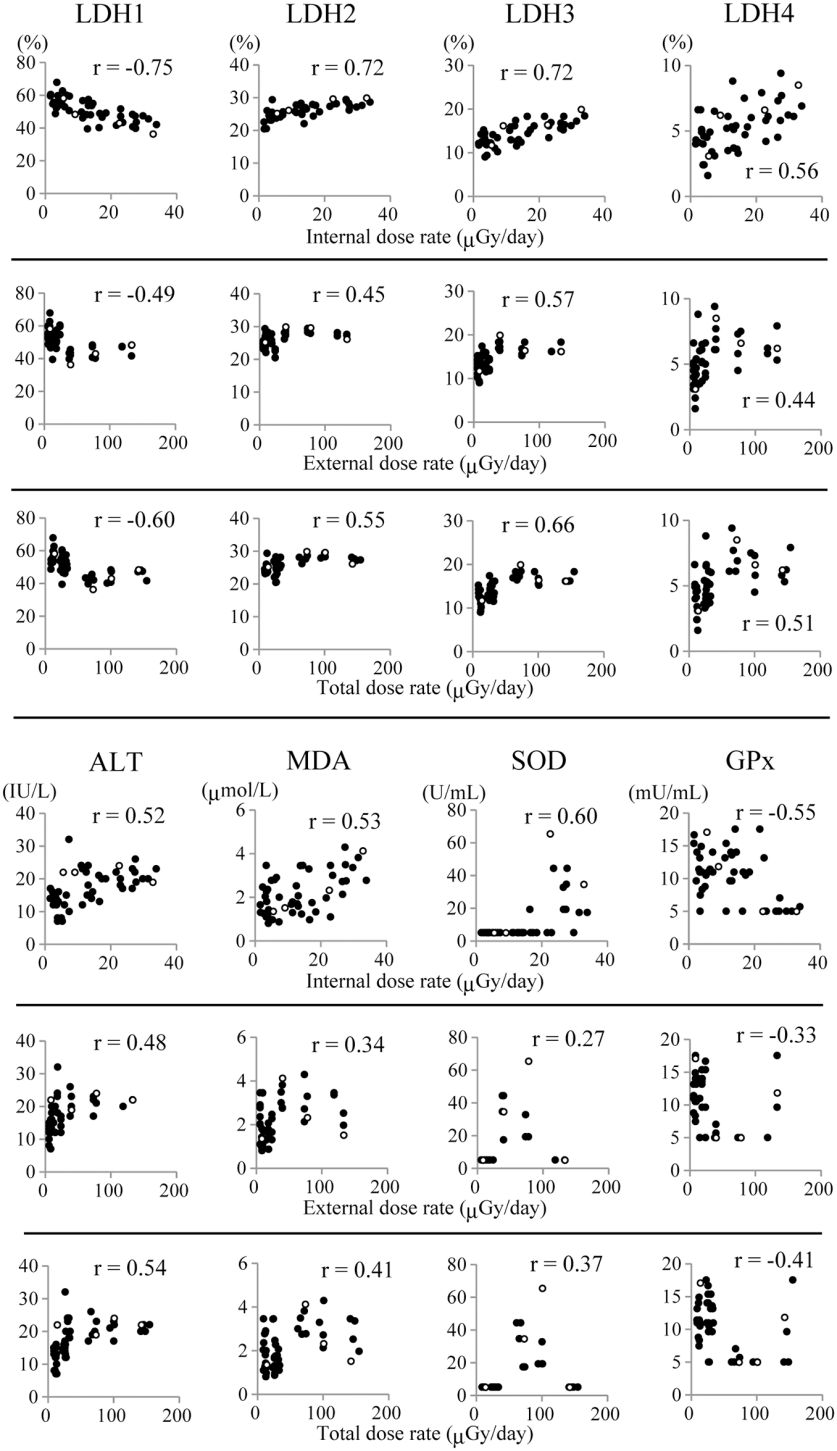
Correlation analysis between dose rate from radiocesium and plasma protein levels. The dot plots indicate the correlation analysis of plasma protein levels with the internal, external or total dose rate in cattle. White circles indicate the values of 4 males and black circles indicate the values of 45 females. r indicates Pearson’s correlation coefficient. p values were Shown in S2 Table.

We analyzed plasma protein concentrations and enzyme activities in relation to each internal, external and total dose rate (Fig 3 and S2 Table). ALT, LDH-2, LDH-3 and LDH-4 were positively, and LDH-1 was negatively correlated with both internal dose rate and total dose rate. LDH-3 was positively correlated with external dose rate. MDA content and SOD activity were positively and GPx activity was negatively correlated with internal dose rate. We could not find particular correlation between oxidative stress markers examined and other plasma components (S3 Table).

For the elucidation of the effect of irradiation on the levels of plasma components, dose response relationship is crucial. Assuming that the cattle in the ex-evacuation zone remained in a small area they were captured, the median of cumulative internal dose was 3.3 mGy (0.3–15.6 mGy) and that of external dose was 4.0 mGy (2.5–76.3 mGy) (S4 Table). Cumulative dose of both internal and external exposure was clearly correlated with dose rate, respectively (r > 0.9, p < 0.01, S3 Fig) Correlation analysis showed that the plasma components correlated with internal dose rate were also well correlated with cumulative internal dose (S5 Table and S4 Fig).

## Discussion

In this study, we performed biochemical analysis of plasma of cattle in the ex-evacuation zone and that in farms of Miyagi prefecture and Yamaguchi prefecture as the control not affected by the FNPP accident. There were significant differences in 10 out of 19 plasma components between Miyagi group and Yamaguchi group, which may be attributable to different breeding conditions (Table 1). TP, ALP and CRE of Miyagi group were significantly different from those of Yamaguchi group, presumably because Miyagi group was before adolescent [24]. Nine out of 19 plasma components in the cattle of the ex-evacuation zone were significantly different from those in Miyagi group and Yamaguchi group. Although, among those 9 plasma components, TP, ALP, CRE and NEFA were not correlated with dose rate, LDH-1, LDH-2, LDH-3, MDA and GPx were correlated with dose rate of the cattle in the ex-evacuation zone. These suggest that the influence of the unleased status, if any, on dose rate and plasma components relationship is negligible.

Plasma LDH-1 was negatively and, ALT, LDH-2, LDH-3 and LDH-4 were positively correlated with both internal and total dose rate from radiocesium (Fig 3). LDH is a tetrameric molecule composed of two different subunits, M and H, which associate to form five isozymes: LDH-1 (H4), LDH-2 (H3M1), LDH-3 (H2M2), LDH-4 (H1M3) and LDH-5 (M4) [25]. LDH-5 is present in glycolytic tissues (e.g., liver and skeletal muscle) and preferentially converts pyruvate to lactate, while LDH-1 is present in aerobic tissues (e.g., the heart) and preferentially drives pyruvate production [26]. LDH-2, LDH-3 and LDH-4 are predominant in the lung and in leukocytes [27]. ALT is well known as an indicator of liver injury [28]. LDH-5 overexpression is associated with radiotherapy resistance in prostate cancer [29]. Therefore, we carried out histological analysis of organs including the liver, skeletal muscle, lung and spleen in the cattle with the 6 highest (H group) and those with the 6 lowest (L group) internal dose rates (S6 Table and S5 Fig). Remarkable histological difference between H group and L group was not observed in all the organs examined. These suggest that LDRR affected the cattle in the ex-evacuation zone systemically but slightly.

In the present study, plasma SOD activity and MDA concentration were positively, and GPx activity was negatively correlated with internal dose rate of radiocesium. The status of oxidative stress markers in radiology staff is contradictory. Compared with the control subjects, catalase activity and MDA level of erythrocytes are significantly lower in the exposed group (0.10–3.86 mGy/month) [14]. The activity of an antioxidant enzyme, paraoxonase (PON1) is lower and MDA level is higher in the serum of radiological department workers (44 workers < 3.5mSv/year and 7 workers over 3.5mSv/year) [30]. Significantly higher serum levels of reactive nitrogen species and MDA are observed in workers of the radiology unit as compared with non-radiology staff [31]. Both lipid oxidation levels and antioxidant thiol groups in plasma from occupationally exposed university hospital staff are significantly higher than non-exposed one [32]. The number of reports on the association of oxidative stress with the Chernobyl accident is limited. The expression levels of inducible nitric oxide synthase, cyclooxygenase 2 and 8-hydroxy-2’-deoxyguanosine, are higher in the bladder epithelium of patients in areas with high levels of radiocontamination (0.4–4.1 μGy/day, calculated from the data in the article) compared with those with low levels [33]. The levels of antioxidants, such as carotenoids and vitamin A and E, in bird blood, liver and egg are lower in Chernobyl (0.390 mR/hour at the ground) compared with uncontaminated control area (0.025 mR/hour at the ground) [34]. In the current study, the median value of total dose rate was 26.9 μGy/day (9.1–155.1 μGy/day) in the cattle in the ex-evacuation zone, which is the same or higher level irradiation compared with the dose rate reported in these previous studies. However, all these previous reports have not carried out dose evaluation of individuals but indicate that LDRR generally increases lipid oxidant levels in spite that antioxidant levels are various. The present study is the first to show relationship between dose-rate of chronic internal exposure and oxidative stress. It has been reported that prior exposure to a low-dose of radiation induces an adaptive response in which cells become less susceptible to a subsequent high dose of radiation [35]. Compared with non-adapted cells, antioxidant enzyme activities increase in adapted cells after a challenging high-dose exposure [36]. Significant overexpression of both SOD and catalase genes has been detected in the spleen cells of mice that were irradiated with gamma rays at 22 mGy/day for 23 days compared with non-irradiated control mice [37]. However, the expression of both SOD and catalase genes reduce to control levels after continued irradiation for further 40 days or 60 days. Therefore, chronic exposure to LDRR might induce an adaptive response that results in negative correlation between internal dose rate and GPx activity in the cattle of the ex-evacuation zone.

The lifespan of female mice exposed to gamma rays at 1.1 mGy/day for 400 days is significantly shorter and tends to be shorter exposed to 0.05 mGy/day for 400 days but not significant compared with non-irradiated controls [38, 39]. These results indicate that long-term LDRR has a very weak biological effect. Chronic exposure to LDRR at the levels of the ex-evacuation zone of FNPP may not induce remarkable biological effects but be enough to induce a slight oxidative stress.

ALT, LDH-1, LDH-2, LDH-3 and LDH-4, were correlated with both internal and total dose rates. Oxidative stress markers such as MDA, and SOD and GPx activities showed correlation with internal dose rate but not with total dose rate (Fig 3). Since this study was not performed under a strictly controlled condition, evaluation of dose rate contains some uncertainties. The cattle could move about within the ex-evacuation zone freely and external dose rate was affected by geological factors and the movement of the cattle. However we assumed that the cattle remained where they were caught until euthanasia. It is reported that the biological half-life of ^137^Cs in the muscle of dairy cow is approximately 30 days [40]. While our preliminary study revealed that the biological half-life of radiocesium in blood is approximately 2 weeks (manuscript in preparation). These results indicate that internal dose rate provides a greater reflection of biologically relevant effects compared with the external exposure in the ex-evacuation zone of FNPP.

The present study suggests that oxidant-related plasma components are associated with long-term exposure to LDRR. However, the main source of the change in the oxidant-related plasma components remains unclear. We previously reported that the radiocesium concentration in organs correlates significantly with that in whole blood [5]. The liver is the largest among the visceral organs, and 1/5 of the total blood travels through the liver in humans and, furthermore, plays the prominent role in the maintenance of plasma homeostasis. However, LDH-5 was not correlated with dose rate. Furthermore, remarkable difference was not observed between the liver of H-group and L-group in the cattle of the ex-evacuation zone examined. MDA, SOD and GPx showed no significant correlation with other plasma components in this study. These suggest that the changes in the levels of the plasma components whose values are different between control groups observed in this study do not affect the correlation of oxidative stress markers with dose rate, and that the level of radiation exposure in the ex-evacuation zone is marginal but causes significant stress without severe organ damages in the cattle. Interestingly it is reported that MDA levels and CuZn-SOD activity are significantly higher and GPx and catalase activities are significantly lower in erythrocytes of patients with oesophageal or gastric cancer than clinically healthy individuals [41]. After the FNPP accident, not only physical but also psychological disturbance has come up as one of subjects of the radiation effect. Furthermore, ROS are postulated to be involved in the process of carcinogenesis [42]. Therefore, we need a long-term vigilant observation of animals including cancer induction in the ex-evacuation zone of FNPP to confirm the radiation effects on the ecosystem and on humans.

## Supporting Information

S1 Fig Representative gamma-spectrometry profile of the skeletal muscle and soil. ^134^Cs and ^137^Cs were vast majority of radionuclides in both skeletal muscle of cattle and soil because 5 months or more had passed since the FNPP accident. Sampling of the skeletal muscle and the soil was performed on January 2012 and July 2012 at the same place in the ex-evacuation zone, respectively.
(PDF)

S2 Fig ^137^Cs concentrations in the peripheral blood and the skeletal muscle of cattle in the ex-evacuation zone. The dot plot shows the correlation of the ^137^Cs concentration of peripheral blood with that of the skeletal muscle in cattle in the ex-evacuation zone. r indicates coefficient of determination. p value is below 0.001.
(PDF)

S3 Fig Correlation analysis between dose rate and cumulative dose of the individual cattle in the ex-evacuation zone. r and p is Pearson’s correlation coefficient and p values, respectively.
(PDF)

S4 Fig Correlation analysis between cumulative dose from radiocesium and plasma component levels in cattle of the ex-evacuation zone. r is Pearson’s correlation coefficient. p values are Shown in S4 Table.
(PDF)

S5 Fig Histological findings observed in each tissue. **a-e**. H&E staining. **f**. Iron staining by Prussian blue staining. **a, b**. *Sarcocystis* cysts in the H1 biceps femoris muscle. **c**. inflammatory cell infiltration in the L6 liver. **d**. inflammatory cell infiltration of kidney in the L2 kidney. **e, f**. Hemosiderin deposition in the L5 Spleen.
(PDF)

S1 Table Radiocesium concentrations in the skeletal muscle of individual cattle and that in the soil where cattle were captured. Numbers in red indicate the values calculated from radiocesium concentration of blood.
(PDF)

S2 Table Correlation coefficient between dose rate and plasma components in cattle of the ex-evacuation zone. r and p is Pearson’s correlation coefficient and p value, respectively.
(PDF)

S3 Table Correlation coefficient between oxidative stress markers and other plasma components in cattle of the ex-evacuation zone. r and p is indicate Pearson’s correlation coefficient and p values, respectively.
(PDF)

S4 Table Cumulative dose of internal and external exposure of radiocesium. Cumulative dose from ^134^Cs and ^137^Cs during certain duration were calculated as a calculation formula written on this Table.
(PDF)

S5 Table Correlation coefficient between cumulative dose and plasma components in cattle of the ex-evacuation zone. r and p is Pearson’s correlation coefficient and p values, respectively.
(PDF)

S6 Table Histological findings in 12 cattle of the ex-evacuation zone. S, *Sarcocystis cyst*; N, no abnormalities; -, no histological analysis; H, Hemosiderin deposition; I, Inflammatory cell infiltration.
(PDF)
