# Correction: Analysis of Plasma Protein Concentrations and Enzyme Activities in Cattle within the Ex-Evacuation Zone of the Fukushima Daiichi Nuclear Plant Accident

**DOI:** 10.1371/journal.pone.0159282

**Published:** 2016-08-08

**Authors:** Yusuke Urushihara, Koh Kawasumi, Satoru Endo, Kenichi Tanaka, Yasuko Hirakawa, Gohei Hayashi, Tsutomu Sekine, Yasushi Kino, Yoshikazu Kuwahara, Masatoshi Suzuki, Motoi Fukumoto, Hideaki Yamashiro, Yasuyuki Abe, Tomokazu Fukuda, Hisashi Shinoda, Emiko Isogai, Toshiro Arai, Manabu Fukumoto

There is an error in title of the article. The correct title should be: Analysis of Plasma Protein Concentrations and Enzyme Activities in Cattle within the Ex-Evacuation Zone of the Fukushima Daiichi Nuclear Power Plant Accident. The correct citation should read as follows: Urushihara Y, Kawasumi K, Endo S, Tanaka K, Hirakawa Y, Hayashi G, et al. (2016) Analysis of Plasma Protein Concentrations and Enzyme Activities in Cattle within the Ex-Evacuation Zone of the Fukushima Daiichi Nuclear Power Plant Accident. PLoS ONE 11(5): e0155069. doi:10.1371/journal.pone.0155069

There is an error in the affiliation for author Emiko Isogai. The correct affiliation should be #10: Graduate School of Agricultural Sciences, Tohoku University, Sendai, Miyagi, Japan.

There is an error in the last sentence of the “Statistical analysis” sub section of the “Materials and Methods” section. The correct sentence should be: Welch’s t test was applied to determine significant differences between 2 groups.

There is an error in Table 1. In the row labeled “Gender” under the column labeled “Ex-evacuation (49)”, the number of females is incorrect. The correct numbers should be: 45.

**Table 1:** Plasma protein concentrations and enzyme activities.

	Ex-evacuation (49)	Miyagi (6)	Yamaguchi (4)
Gender	45 females and 4 males^#^	6 males^##^	1 female and 3 males^##^
Age (year)*	6.36 ± 3.26^†^	0.69 ± 0.02	1.71 ± 0.21
TP (g/dL)*	6.96 ± 0.85^†^	6.23 ± 0.43	7.40 ± 0.28
TG (mg/dL)	13.6 ± 9.7	11.3 ± 3.1	19.3 ± 5.4
AST (IU/L)	72.9 ± 24.7	55.8 ± 5.9	86.8 ± 21.9
ALT (IU/L)	16.5 ± 5.8	23.3 ± 2.1	22.8 ± 5.1
ALP (IU/L)*	157.0 ± 122.9^†^	571.5 ± 192.3	284.0 ± 76.6
LDH (IU/L)*	1006.1 ± 256.1	953.5 ± 66.4	1492.3 ± 113.5
LDH-1 (%)*	50.8 ± 7.4^†^	41.6 ± 0.8	47.2 ± 1.6
LDH-2 (%)	25.7 ± 2.4^†^	29.3 ± 1.1	28.6 ± 1.2
LDH-3 (%)*	14.5 ± 3.4^†^	18.6 ± 0.6	16.8 ± 0.7
LDH-4 (%)*	5.3 ± 1.8	6.6 ± 0.6	4.8 ± 0.9
LDH-5 (%)	3.9 ± 2.0	4.0 ± 0.9	2.7 ± 1.1
BUN (mg/dL)	8.0 ± 4.9	12.5 ± 2.1	12.3 ± 4.9
CRE (mg/dL)*	1.34 ± 0.26^†^	0.78 ± 0.10	1.2 ± 0.00
TC (mg/dL)*	91.5 ± 35.9	78.0 ± 20.5	125.5 ± 26.0
GLU (mg/dL)	88.1 ± 67.0	89.8 ± 4.7	103.0 ± 46.3
NEFA (μEq/L)	308 ± 192^†^	140 ± 79	87 ± 5
MDA (μmol/L)*	2.19 ± 0.94^†^	0.83 ± 0.09	1.08 ± 0.13
SOD (U/mL)	12.7 ± 14.1	45.0 ± 34.3	25.8 ± 6.7
GPx (mU/mL)*	10.4 ± 3.9^†^	6.4 ± 3.5	51.6 ± 9.5

The numbers in parentheses indicate the number of animals examined.

^#^Two were castrated.

^##^All the males were castrated.

*Significantly different between Miyagi and Yamaguchi groups (p < 0.05).

^†^Significantly higher or lower than both Miyagi and Yamaguchi groups (p < 0.05).
