# Supplementary material for: Analysis of Plasma Protein Concentrations and Enzyme Activities in Cattle within the Ex-Evacuation Zone of the Fukushima Daiichi Nuclear Plant Accident
Source: PLoS One. 2016 May 9;11(5):e0155069. doi: 10.1371/journal.pone.0155069 (PMC4861266; doi:10.1371/journal.pone.0155069)
Supplement: S1 Fig — 134Cs and 137Cs were vast majority of radionuclides in both skeletal muscle of cattle and soil because 5 months or more had passed since the FNPP accident. Sampling of the skeletal muscle and the soil was performed on January 2012 and July 2012 at the same place in the ex-evacuation zone, respectively. (PDF) [file pone.0155069.s001.pdf]

S1 Fig. Representative gamma-spectrometry profile of the skeletal muscle and soil

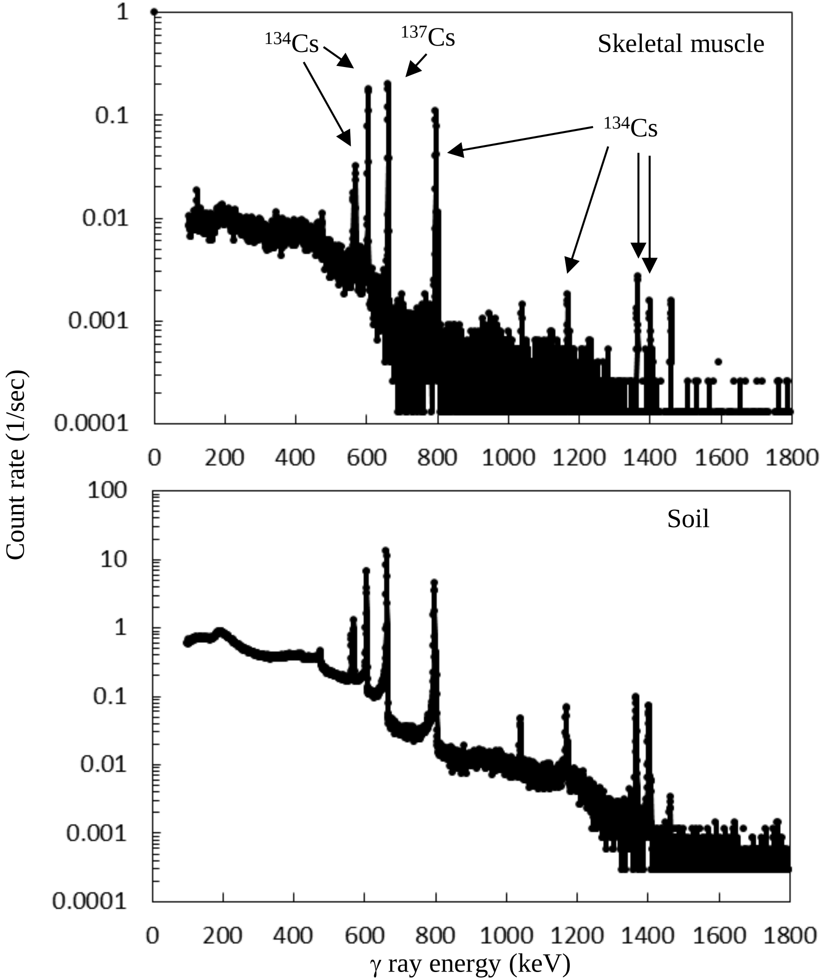

^{134}Cs and ^{137}Cs were vast majority of radionuclides in both skeletal muscle of cattle and soil because 5 months or more had passed since the FNPP accident. Sampling of the skeletal muscle and the soil was performed on January 2012 and July 2012 at the same place in the ex-evacuation zone, respectively.
