# Supplementary material for: Analysis of Plasma Protein Concentrations and Enzyme Activities in Cattle within the Ex-Evacuation Zone of the Fukushima Daiichi Nuclear Plant Accident
Source: PLoS One. 2016 May 9;11(5):e0155069. doi: 10.1371/journal.pone.0155069 (PMC4861266; doi:10.1371/journal.pone.0155069)
Supplement: S2 Fig — The dot plot shows the correlation of the 137Cs concentration of peripheral blood with that of the skeletal muscle in cattle in the ex-evacuation zone. r indicates coefficient of determination. p value is below 0.001. (PDF) [file pone.0155069.s002.pdf]

S2 Fig. ^{137}Cs concentrations in the peripheral blood and the skeletal muscle of cattle in the ex-evacuation zone

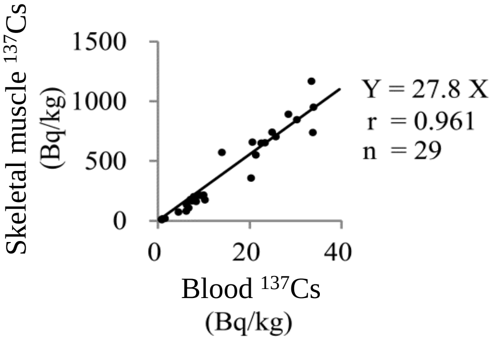

The dot plot shows the correlation of the ^{137}Cs concentration of peripheral blood with that of the skeletal muscle in cattle in the ex-evacuation zone. r indicates coefficient of determination. p value < 0.001.
