# Supplementary material for: Analysis of Plasma Protein Concentrations and Enzyme Activities in Cattle within the Ex-Evacuation Zone of the Fukushima Daiichi Nuclear Plant Accident
Source: PLoS One. 2016 May 9;11(5):e0155069. doi: 10.1371/journal.pone.0155069 (PMC4861266; doi:10.1371/journal.pone.0155069)
Supplement: S3 Fig — r and p is Pearson’s correlation coefficient and p values, respectively. (PDF) [file pone.0155069.s003.pdf]

S3 Fig. Correlation analysis between dose rate and cumulative dose of the individual cattle in the ex-evacuation zone

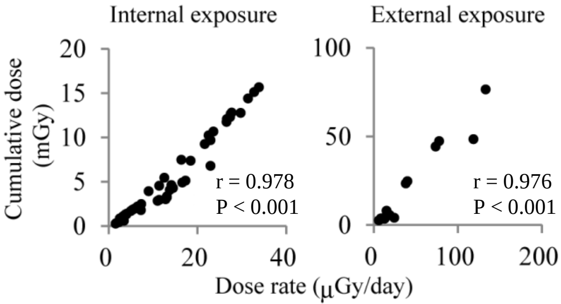

r and p is Pearson's correlation coefficient and p values, respectively.
