# Supplementary material for: Analysis of Plasma Protein Concentrations and Enzyme Activities in Cattle within the Ex-Evacuation Zone of the Fukushima Daiichi Nuclear Plant Accident
Source: PLoS One. 2016 May 9;11(5):e0155069. doi: 10.1371/journal.pone.0155069 (PMC4861266; doi:10.1371/journal.pone.0155069)
Supplement: S4 Fig — r is Pearson’s correlation coefficient. p values are Shown in S4 Table. (PDF) [file pone.0155069.s004.pdf]

**S4 Fig. Correlation analysis between cumulative dose from radiocesium and plasma component levels in cattle of the ex-evacuation zone**

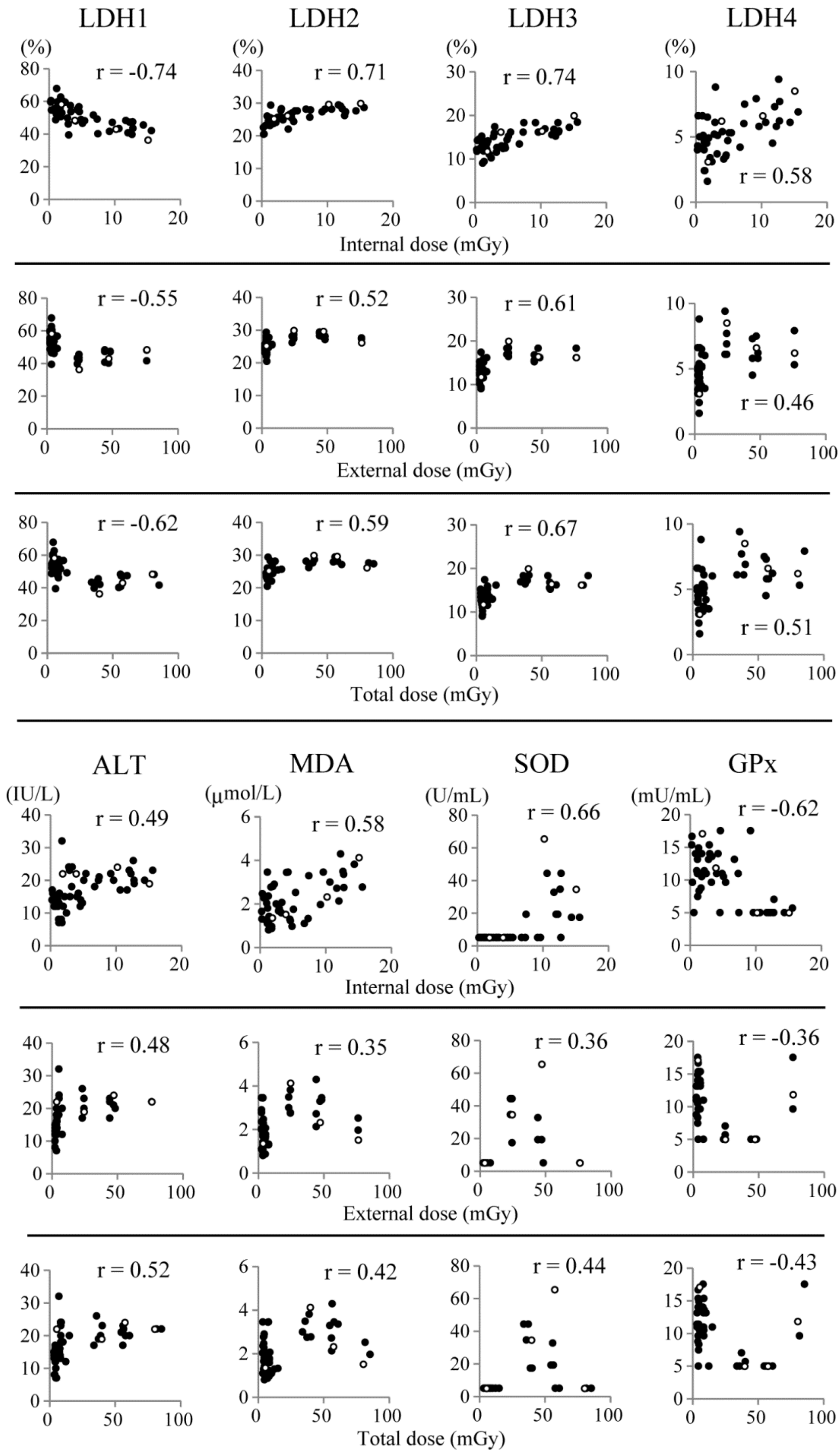


r is Pearson's correlation coefficient. p values are Shown in S4 Table.
