# Supplementary material for: Analysis of Plasma Protein Concentrations and Enzyme Activities in Cattle within the Ex-Evacuation Zone of the Fukushima Daiichi Nuclear Plant Accident
Source: PLoS One. 2016 May 9;11(5):e0155069. doi: 10.1371/journal.pone.0155069 (PMC4861266; doi:10.1371/journal.pone.0155069)
Supplement: S5 Fig — a-e. H&E staining. f. Iron staining by Prussian blue staining. a, b. Sarcocystis cysts in the H1 biceps femoris muscle. c. inflammatory cell infiltration in the L6 liver. d. inflammatory cell infiltration of kidney in the L2 kidney. e, f. Hemosiderin deposition in the L5 Spleen. (PDF) [file pone.0155069.s005.pdf]

S5 Fig. Histological findings observed in each tissue

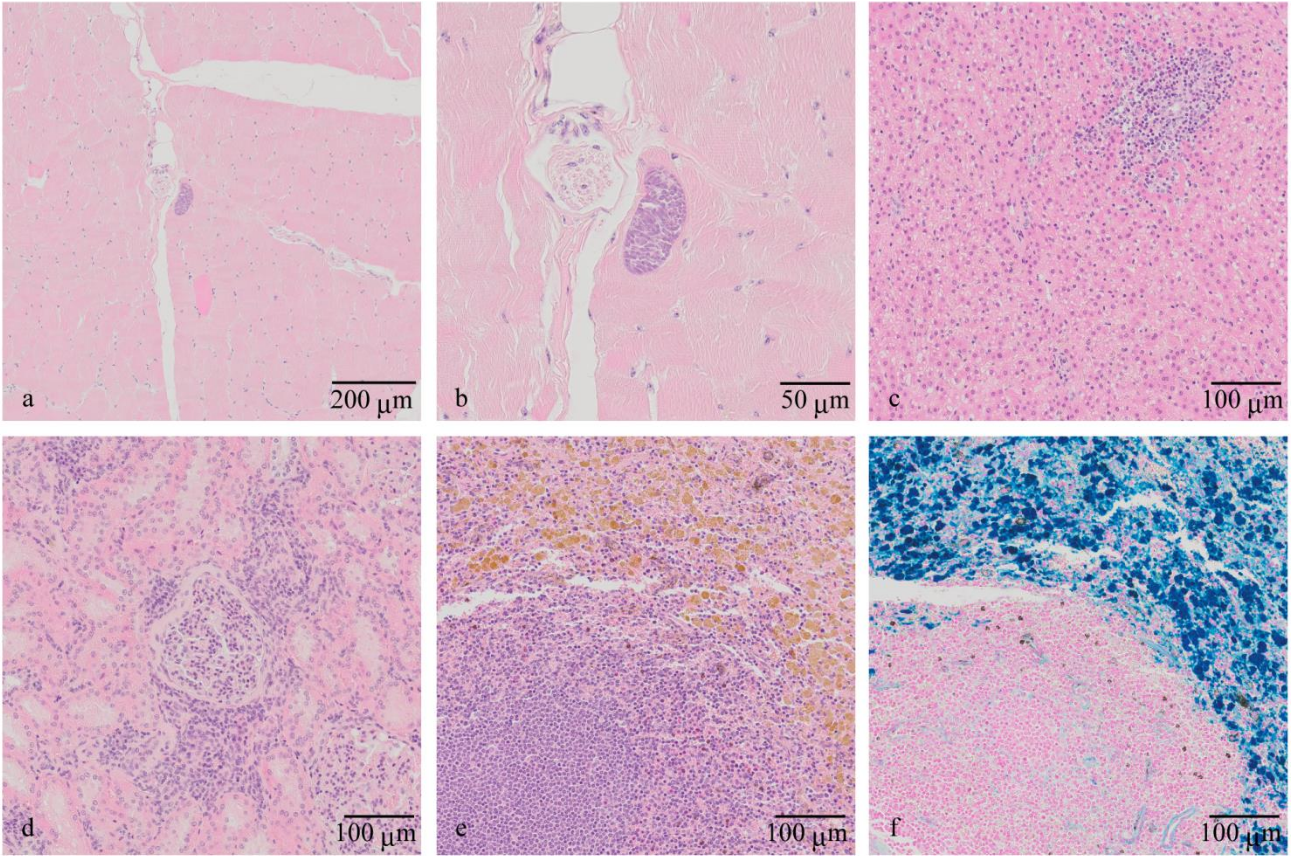

a-e. H&E staining. **f**. Iron staining by Prussian blue staining. **a, b.** *Sarcocystis* cysts in the H1 biceps femoris muscle. **c.** inflammatory cell infiltration in the L6 liver. **d.** inflammatory cell infiltration of kidney in the L2 kidney. **e, f.** Hemosiderin deposition in the L5 Spleen.
