# Supplementary material for: Analysis of Plasma Protein Concentrations and Enzyme Activities in Cattle within the Ex-Evacuation Zone of the Fukushima Daiichi Nuclear Plant Accident
Source: PLoS One. 2016 May 9;11(5):e0155069. doi: 10.1371/journal.pone.0155069 (PMC4861266; doi:10.1371/journal.pone.0155069)
Supplement: S1 Table — Numbers in red indicate the values calculated from radiocesium concentration of blood. (PDF) [file pone.0155069.s006.pdf]

S1 Table. Radiocesium concentrations in the skeletal muscle of individual cattle and that in the soil where cattle were captured

No.	Sampling date	Radiocesium concentration			
		Skeletal muscle Bq/kg		Soil Bq/m^2	
		^{137}Cs	^{134}Cs	^{137}Cs	^{134}Cs
1	2011/8/31	74.1	63.7	297014.1	247585.6
2	2011/8/31	165.6	141.5	297014.1	247585.6
3	2011/8/31	81.2	68.5	297014.1	247585.6
4	2011/8/31	107.7	94.1	297014.1	247585.6
5	2011/11/15	649.9	503.7	183573.1	146166.3
6	2011/11/29	652.8	521.7	269555.2	213646.4
7	2011/11/29	357.9	289.8	269555.2	213646.4
8	2011/12/9	572.1	450.9	269384.9	211687.3
9	2011/12/9	549.8	437.5	269384.9	211687.3
10	2011/12/9	658.1	516.2	269384.9	211687.3
11	2012/1/24	1167.9	895.6	152825	109983.3
12	2012/1/24	846.1	643.4	152825	109983.3
13	2012/1/24	890.6	669.7	152825	109983.3
14	2012/1/24	703.7	535.0	152825	109983.3
15	2012/1/24	739.6	562.8	152825	109983.3
16	2012/3/1	133.5	97.1	136426.6	95518.23
17	2012/3/1	213.1	153.3	136426.6	95518.23
18	2012/3/1	185.6	133.8	136426.6	95518.23
19	2012/3/1	741.5	539.6	136426.6	95518.23
20	2012/3/8	418.3	260.6	91809.3	65520.13
21	2012/3/8	345.2	245.1	91809.3	65520.13
22	2012/3/8	272.6	184.2	91809.3	65520.13
23	2012/3/8	162.7	117.3	91809.3	65520.13
24	2012/3/8	177.8	128.1	91809.3	65520.13
25	2012/3/8	309.1	226.4	91809.3	65520.13
26	2012/3/8	173.9	132.2	91809.3	65520.13
27	2012/3/8	207	140	91809.3	65520.13
28	2012/3/14	200	145	133664.3	91386.53
29	2012/3/14	291.8	206.1	133664.3	91386.53
30	2012/3/14	263	208	133664.3	91386.53
31	2012/3/14	213.9	156.5	133664.3	91386.53
32	2012/5/25	638.3	413.8	244872	164345.3
33	2012/5/25	1032.9	671.10	244872	164345.3
34	2012/6/27	1864.4	967.7	1382707	926691.9
35	2012/6/27	1201.1	873.3	1382707	926691.9
36	2012/7/10	488.1	334.5	2207312	1390773
37	2012/7/10	679.8	464.7	2207312	1390773
38	2012/7/10	950.3	926.6	2207312	1390773
39	2012/7/31	1177.0	1129.4	1228569	763901.4
40	2012/7/31	1475.5	1020.7	1228569	763901.4
41	2012/7/31	1534.8	939.6	1228569	763901.4
42	2012/8/3	947.2	575.1	1306851	808743.3
43	2012/8/3	1245.3	824.0	1306851	808743.3
44	2012/8/7	1199.9	917.2	626551.8	400780.7
45	2012/8/7	1643.5	938.9	626551.8	400780.7
46	2012/8/10	1928.2	1130.9	671849.8	414980.9
47	2012/8/10	1648.6	949.5	671849.8	414980.9
48	2012/8/10	1742.0	1142.8	671849.8	414980.9
49	2012/8/10	2046.4	1139.0	671849.8	414980.9

Numbers in red indicate the values calculated from radiocesium concentration of blood.
