# Supplementary material for: Analysis of Plasma Protein Concentrations and Enzyme Activities in Cattle within the Ex-Evacuation Zone of the Fukushima Daiichi Nuclear Plant Accident
Source: PLoS One. 2016 May 9;11(5):e0155069. doi: 10.1371/journal.pone.0155069 (PMC4861266; doi:10.1371/journal.pone.0155069)
Supplement: S2 Table — r and p is Pearson’s correlation coefficient and p value, respectively. (PDF) [file pone.0155069.s007.pdf]

S2 Table. Correlation coefficient between dose rate and plasma components in cattle of the ex-evacuation zone

	Internal dose rate		External dose rate		Total dose rate	
	r	P	r	p	r	p
TP	0.22	0.17	0.07	0.64	0.11	0.47
AST	0.28	0.06	0.19	0.22	0.23	0.14
TG	0.05	0.73	-0.03	0.86	-0.01	0.94
ALT	0.53	< 0.01	0.48	< 0.01	0.54	< 0.01
ALP	-0.06	0.71	0.00	0.99	-0.01	0.94
LDH	0.26	0.09	0.09	0.55	0.14	0.37
LDH-1	-0.75	< 0.01	-0.49	< 0.01	-0.60	< 0.01
LDH-2	0.72	< 0.01	0.45	< 0.01	0.55	< 0.01
LDH-3	0.72	< 0.01	0.57	< 0.01	0.66	< 0.01
LDH-4	0.56	< 0.01	0.44	< 0.01	0.51	< 0.01
LDH-5	0.30	0.05	0.04	0.80	0.10	0.52
BUN	0.17	0.27	-0.31	0.04	-0.23	0.13
CRE	-0.33	0.03	-0.22	0.16	-0.26	0.09
TC	-0.10	0.54	-0.26	0.09	-0.25	0.11
GLU	0.35	0.02	-0.16	0.32	-0.06	0.72
NEFA	0.05	0.77	0.06	0.71	0.06	0.67
MDA	0.53	< 0.01	0.34	0.03	0.41	< 0.01
SOD	0.60	< 0.01	0.27	0.08	0.37	0.02
GPx	-0.55	< 0.01	-0.33	0.03	-0.41	< 0.01

r and p is Pearson's correlation coefficient and p value, respectively.
