# Supplementary material for: Analysis of Plasma Protein Concentrations and Enzyme Activities in Cattle within the Ex-Evacuation Zone of the Fukushima Daiichi Nuclear Plant Accident
Source: PLoS One. 2016 May 9;11(5):e0155069. doi: 10.1371/journal.pone.0155069 (PMC4861266; doi:10.1371/journal.pone.0155069)
Supplement: S3 Table — r and p is indicate Pearson’s correlation coefficient and p values, respectively. (PDF) [file pone.0155069.s008.pdf]

S3 Table. Correlation coefficient between oxidative stress markers and other plasma components in cattle of the ex-evacuation zone

	MDA		SOD		GPx	
	r	p	r	p	r	p
TP	0.24	0.12	0.46	< 0.01	-0.17	0.27
AST	-0.02	0.89	0.07	0.64	0.11	0.47
TG	0.36	0.06	0.27	0.08	-0.35	0.02
ALT	0.19	0.23	0.32	0.04	-0.11	0.48
ALP	-0.19	0.22	-0.06	0.70	0.31	0.04
LDH	0.07	0.65	0.27	0.08	-0.09	0.56
LDH-1	-0.47	< 0.01	-0.54	< 0.01	0.39	0.01
LDH-2	0.26	0.09	0.51	< 0.01	-0.39	0.01
LDH-3	0.47	< 0.01	0.50	< 0.01	-0.40	< 0.01
LDH-4	0.42	< 0.01	0.46	< 0.01	-0.34	0.02
LDH-5	0.29	0.06	0.23	0.13	-0.15	0.32
BUN	0.15	0.35	0.05	0.77	0.02	0.88
CRE	-0.22	0.16	-0.23	0.14	0.21	0.17
TC	-0.12	0.43	-0.07	0.64	0.18	0.25
GLU	0.01	0.97	0.21	0.18	0.03	0.83
NEFA	0.04	0.78	-0.15	0.32	-0.20	0.20

r and p is Pearson's correlation coefficient and p values, respectively.
