# Supplementary material for: Analysis of Plasma Protein Concentrations and Enzyme Activities in Cattle within the Ex-Evacuation Zone of the Fukushima Daiichi Nuclear Plant Accident
Source: PLoS One. 2016 May 9;11(5):e0155069. doi: 10.1371/journal.pone.0155069 (PMC4861266; doi:10.1371/journal.pone.0155069)
Supplement: S4 Table — Cumulative dose from 134Cs and 137Cs during certain duration were calculated as a calculation formula written on this Table. (PDF) [file pone.0155069.s009.pdf]

S4 Table. Cumulative dose of internal and external exposure of radiocesium

Cumulative dose (mGy)*	Mean	Median	Range
Internal exposure	5.3 ± 4.7	3.3	0.3 – 15.6
External exposure	16.9 ± 21.7	4.0	2.5 – 76.3
Total exposure	22.1 ± 24.8	8.5	3.5 – 85.5

*The cumulative dose from ^{134}Cs and ^{137}Cs during certain duration were calculated as

$$\sum_{k=0}^{n} \{C_{Cs134} A_{Cs134} \frac{1}{2}^{\frac{k}{T_{1/2\,Cs134}}} + C_{Cs137} A_{Cs137} \left(\frac{1}{2}\right)^{\frac{k}{T_{1/2\,Cs137}}}\}$$

C; coefficient (μGy/day)/(Bq/kg) for internal exposure, (μGy/day)/(Bq/m^2) for external exposure of the reference deer by ICRP Pub.108.

A; radiocesium concentration (Bq/kg) in the skeletal muscle for internal exposure or soil (Bq/m^2) for external exposure as of March 15, 2011.

$T_{1/2}$; half life (days), 754 for ^{134}Cs and 10983 for ^{137}Cs.

n; period from March 15 to the day of capture (days).
