# Supplementary material for: Analysis of Plasma Protein Concentrations and Enzyme Activities in Cattle within the Ex-Evacuation Zone of the Fukushima Daiichi Nuclear Plant Accident
Source: PLoS One. 2016 May 9;11(5):e0155069. doi: 10.1371/journal.pone.0155069 (PMC4861266; doi:10.1371/journal.pone.0155069)
Supplement: S5 Table — r and p is Pearson’s correlation coefficient and p values, respectively. (PDF) [file pone.0155069.s010.pdf]

S5 Table. Correlation coefficient between cumulative dose and plasma components in cattle of the ex-evacuation zone

	Internal dose		External dose		Total dose	
	r	p	r	p	r	p
TP	0.26	0.9	0.11	0.48	0.15	0.35
AST	0.23	0.15	0.19	0.23	0.21	0.18
TG	0.15	0.33	0.06	0.71	0.08	0.61
ALT	0.49	< 0.01	0.48	< 0.01	0.52	< 0.01
ALP	-0.07	0.67	0.00	0.99	-0.01	0.94
LDH	0.24	0.12	0.17	0.28	0.19	0.22
LDH-1	-0.74	< 0.01	-0.55	< 0.01	-0.62	< 0.01
LDH-2	0.71	< 0.01	0.52	< 0.01	0.59	< 0.01
LDH-3	0.74	< 0.01	0.61	< 0.01	0.67	< 0.01
LDH-4	0.58	< 0.01	0.46	< 0.01	0.51	< 0.01
LDH-5	0.25	0.10	0.08	0.62	0.11	0.46
BUN	0.08	0.63	-0.32	0.04	-0.27	0.08
CRE	-0.30	0.05	-0.18	0.26	-0.21	0.17
TC	-0.18	0.24	-0.28	0.07	-0.28	0.07
GLU	0.26	0.09	-0.11	0.49	-0.05	0.77
NEFA	-0.02	0.90	-0.01	0.93	-0.02	0.92
MDA	0.58	< 0.01	0.35	0.02	0.42	< 0.01
SOD	0.66	< 0.01	0.36	0.02	0.44	< 0.01
GPx	-0.62	< 0.01	-0.36	0.02	-0.43	< 0.01

r and p is Pearson's correlation coefficient and p values, respectively.
