# Supplementary material for: Analysis of Plasma Protein Concentrations and Enzyme Activities in Cattle within the Ex-Evacuation Zone of the Fukushima Daiichi Nuclear Plant Accident
Source: PLoS One. 2016 May 9;11(5):e0155069. doi: 10.1371/journal.pone.0155069 (PMC4861266; doi:10.1371/journal.pone.0155069)
Supplement: S6 Table — S, Sarcocystis cyst; N, no abnormalities; -, no histological analysis; H, Hemosiderin deposition; I, Inflammatory cell infiltration. (PDF) [file pone.0155069.s011.pdf]

S6 Table. Histological findings in 12 cattle of the ex-evacuation zone

Sample No.	Dose rate (μGy/day)			Abnormal findings				
	Internal	External	Total	Muscle	Lung	Liver	Kidney	Spleen
H1	33.9	40.1	74.0	S	N	-	N	-
H2	32.8	40.1	72.9	S	-	N	-	H
H3	31.4	40.1	71.5	S	N	N	N	H
H4	27.6	38.2	65.8	S	N	N	N	-
H5	26.7	73.6	100.3	S	N	N	N	I
L1	4.0	8.8	12.9	S	N	-	I	N
L2	3.5	8.8	12.4	N	N	N	N	H
L3	3.4	6.0	9.4	S	N	N	N	N
L4	3.1	6.0	9.1	S	N	N	N	N
L5	2.6	8.8	11.4	S	N	N	N	H

S, *Sarcocystis cyst*; N, no abnormalities; -, no histological analysis; H, Hemosiderin deposition; I, Inflammatory cell infiltration.
